# Towards More Sustainable Material Formulations: A Comparative Assessment of PA11-SGW Flexural Performance versus Oil-Based Composites

**DOI:** 10.3390/polym10040440

**Published:** 2018-04-14

**Authors:** Helena Oliver-Ortega, José Alberto Méndez, Rafel Reixach, Francesc Xavier Espinach, Mònica Ardanuy, Pere Mutjé

**Affiliations:** 1 Group LEPAMAP, Department of Chemical Engineering, University of Girona, C/M. Aurèlia Capmany, 61, 17003 Girona, Spain; jalberto.mendez@udg.edu (J.A.M.); pere.mutje@udg.edu (P.M.); 2 Department of Architecture and Construction Engineering, University of Girona, C/M. Aurèlia Capmany, 61, 17003, Girona, Spain; rafel.reixach@udg.edu; 3 Design, Development and Product Innovation, Department Organization, Business Management and Product Design, University of Girona, C/M. Aurèlia Capmany, 61, 17003 Girona, Spain; francisco.espinach@udg.edu; 4 Departament de Ciència dels Materials i Enginyeria Metal·lúrgica, Secció Enginyeria Tèxtil, Universitat Politècnica de Catalunya, C/Colom, 11, 08222 Terrassa, Barcelona, Spain; monica.ardanuy@upc.edu

**Keywords:** flexural properties, polyamide 11, lignocellulosic fibers, polypropylene composites, fiber/matrix bond

## Abstract

The replacement of commodity polyolefin, reinforced with glass fiber (GF), by greener alternatives has been a topic of research in recent years. Cellulose fibers have shown, under certain conditions, enough tensile capacities to replace GF, achieving competitive mechanical properties. However, if the objective is the production of environmentally friendlier composites, it is necessary to replace oil-derived polymer matrices by bio-based or biodegradable ones, depending on the application. Polyamide 11 (PA11) is a totally bio-based polyamide that can be reinforced with cellulosic fibers. Composites based on this polymer have demonstrated enough tensile strength, as well as stiffness, to replace GF-reinforced polypropylene (PP). However, flexural properties are of high interest for engineering applications. Due to the specific character of short-fiber-reinforced composites, significant differences are expected between the tensile and flexural properties. These differences encourage the study of the flexural properties of a material prior to the design or development of a new product. Despite the importance of the flexural strength, there are few works devoted to its study in the case of PA11-based composites. In this work, an in-depth study of the flexural strength of PA11 composites, reinforced with Stoneground wood (SGW) from softwood, is presented. Additionally, the results are compared with those of PP-based composites. The results showed that the SGW fibers had lower strengthening capacity reinforcing PA11 than PP. Moreover, the flexural strength of PA11-SGW composites was similar to that of PP-GF composites.

## 1. Introduction

Flexural strength and strain are of great interest in the design and product development fields [1,2,3,4,5]. In short-fiber-reinforced composite materials, the in-depth study of these properties is relevant to the different fiber orientations in the skin, the shell, and the core of injected specimens regarding the load axis [5]. Consequently, a significant gap between the tensile and flexural properties of such composites is expected [6,7]. From an engineering point of view, depending on constraints and loads applied to a geometry, the failure point will be defined by the tensile and flexural properties of the material [8]. In addition, one of the most common applications for wood polymer composites are decking profiles, supporting the flexural loads between the points that hold them [5].

**Figure 1:**
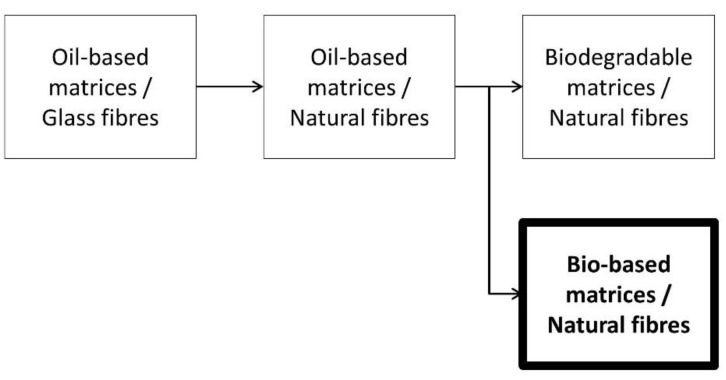
Evolution of composite materials in recent years.

A schematic evolution of composite materials from synthetic fibers, such as glass fibers (GF), to greener materials, is represented in Figure 1.

In view of environmental awareness, one of the goals of this evolution is the production of totally bio-based and biodegradable composites. GF has been one of the most often used reinforcements for petroleum-based matrices, due to its high capacity to increase the strength and modulus of the resulting composite materials [1], combined with a low production cost and availability. Nowadays, 98% of the whole composites’ production in Europe are reinforced with GF [9,10]. However, one of the main drawbacks of the use of GF is related to its high stiffness, which leads to a drop of the toughness of the materials, giving rise to a reduction of the recyclability of these materials [11]. This reduced recyclability does not agree with the European Union (EU) solid waste treatment targets for 2025 and 2030 related to the improvement of the performance of recycled plastics [12]. In this sense, many tons of GF waste from composite materials are deposited in landfills [9]. Otherwise, another disadvantage of the use of GF is related to its health risks, as dermatitis and respiratory diseases can be caused by the manipulation of this material [13,14].

In order to avoid these drawbacks derived from the use of GF, efforts have been devoted to substitute GF by environmentally friendlier reinforcements. An example of these greener reinforcements are cellulosic or lignocellulosic fibers, which have successfully replaced GF as reinforcement for oil-based polymers in some industrial fields such as automotive, construction, and clinical fields [15,16,17]. Cellulose is a natural and sustainable polymer that has the potential to become a raw material for energy production (incineration) and a source of reinforcement for polymer matrices [1,5,7,18].

Despite their availability and widespread use in the industry, the use of oil-based polymers, such as polypropylene (PP), as the matrix in composite materials is not desirable. In recent years, research centers and actors in industry have focused on the research and development of more sustainable materials using biopolymers as matrices, such as thermoplastic starch [19] or polylactic acid (PLA) [20]. Nonetheless, the relatively fast biodegradability of these materials becomes a drawback for their long-term applications, being considered as a greener solution. One promising example of bio-based matrices are bio-polyamides (BioPA). Polyamides (PA), including BioPA and oil-based PA, show good mechanical and insulation properties as well as thermal resistance [21,22]. Moreover, depending on their chemical structure and the raw materials required for their production, BioPA can be totally or partially bio-based. Some examples are polyamide 11 (PA11), which is 100% bio-based, polyamide 10.10 (PA10.10), which is up to 99% bio-based, or polyamide 6.10 (PA6.10), which only contains 62% of carbon from renewable resources [23]. Besides, PA overcomes the problems related to the hydrophobic nature of PP, which penalizes the quality of its interface with hydrophilic reinforcements [24,25,26,27]. The hydrophilic behavior of polyamide matrices [28,29] allows their reinforcement with natural fibers and the achievement of significant improvements to their mechanical properties without the use of coupling agents [23,30,31,32]. Moreover, PA11, which is 100% bio-based (obtained from castor oil) and non-biodegradable, shows a low melting temperature when compared with other PAs [11,33]. This phenomenon is very interesting when cellulose fibers are considered as reinforcement due to their relatively low decomposition temperature (*T* ≈ 200 °C) [34,35].

Stoneground wood (SGW) from pine is a commercially and sustainable fiber produced for the paper industry. Its low cost, high-yield process, and continuous production made it an interesting alternative to GF as a composite reinforcement [36,37]. These fibers have been widely researched as a composite reinforcement, and the mechanical properties of SGW-based composites reveal its competitiveness. According to the literature, the intrinsic tensile strength of SGW in the case of PP-SGW coupled composites is 618 MPa [36]. The presence of coupling agents guarantees a well-bonded system. A slight difference was obtained when the SGW tensile strength was calculated for PA11-SGW composites, which was found to be 562 MPa [38].

The literature on natural fiber-reinforced PA11 composites is scarce. The natural fibers used in such researches are mainly short wood fibers like pine, beech, or commercial pulps and injection molding processes [35,38,39,40,41,42]. The tensile properties obtained with chemically modified beech fiber-reinforced PA11 composites were similar to those of SGW-reinforced composites [35,38]. Nonetheless, the chemical treatment of the beech fibers can increase the cost of the composites compared to those that employ SGW. Armioun, Shaghayegh et al. obtained lower results with commercial wood fiber-reinforced PA11. The lower results were probably due to processing parameters that allowed the presence of noticeable percentages of voids inside the composite. Other works used flax fibers and flax tape [34,43]. These studies obtained high tensile strengths, but they used long aligned fibers and molding press processes. Anyhow, such properties are difficult to obtain with mold injected specimens. Additionally, flax is a comparatively expensive source for reinforcing fibers. Only Armioun, Shaghayegh et al. researched the flexural properties of the composites [41]. Thus, to the best knowledge of the authors, the literature devoted to the flexural properties of PA11-natural fiber composites is scarce.

The present work provides an in-depth analysis of the flexural strength and deformation of SGW-reinforced PA11 composites. Five different materials with SGW contents, ranging from 20% to 60% (*w*/*w*), were prepared by injection-molding and characterized to obtain their experimental flexural performance. These results were compared with the tensile properties obtained in previous studies [38,44]. Then, a fiber flexural strength factor was used to study the neat contribution of the fibers to the flexural strength and such values were compared to their tensile counterparts. Micromechanics models were used to compute the intrinsic flexural strength and modulus of the reinforcement. Finally, the flexural properties of the PA11-based materials were compared with those of GF-reinforced PP commercial composites.

## 2. Materials and Methods

### 2.1. Materials

Polyamide 11 (PA11) (Rilsan^®^ BMNO TL, Colombes, France) was used as a polymer matrix, kindly supplied by Arkema S.A (Colombes, France), with a density of 1.030 g/cm^3^ and a melting temperature around 189 °C.

Stoneground wood (SGW) was used as lignocellulosic reinforcement. SGW was derived from softwood (*Pinus radiata*) and supplied by Zubialde, S.A. (Aizarnazabal, Spain).

### 2.2. Composite Compounding and Sample Obtaining

PA11 was reinforced with five different fiber contents, ranging from 20 up to 60% *w*/*w* of SGW. The compounding process was performed using a Gelimat kinetic mixer (model G5S, Draiswerke, Mahaw, NJ, USA). PA11 and SGW were added at a low speed (300 rpm) and then the speed was raised up to 2500 rpm. The polymeric phase blended when it reached 200 °C, after which the blend was discharged. The extraction of the composite from the kinetic mixer was achieved by gravity discharge.

Afterwards, all of the blends were pelletized by means of a mill equipped with a set of blades. The obtained blends were injected into a Meteor-40 injection machine (Mateu and Solé, Barcelona, Spain; clamping pressure: 40 tons) to obtain the standard specimens for the bending test (ASTM D3641). The samples were conditioned in a climatic chamber at 23 °C and 50% Relative Humidity (RH) before the mechanical test in accordance with ASTM D618.

### 2.3. Mechanical Characterization

Composites were tested under three-point bend configuration in accordance with ASTM D790 standard specifications, using a Universal testing machine supplied by IDMtest (Instron^TM^ 1122, Mark-10 Corporation, Copiague, New York, NY, USA), equipped with a 5-kN load cell. Flexural strength and deformation at the maximum load were obtained from an average of at least five samples.

### 2.4. Composite Density Determination

The composite’s density (*ρ^C^*) was obtained using a pycnometer. A certain weight of the composite was introduced into the pycnometer and the pycnometer was raised to the calibrated volume with distilled water. The density was calculated using the following equation:

(1) $$\rho^{C}=\frac{Weight_{\mathrm{composite}}}{V_{\mathrm{total}}-Weight_{\mathrm{water}}\cdot {\left(\rho_{\mathrm{water}}\right)}^{-1}}$$

where *V*_total_ is the total volume of the pycnometer, and *ρ*_water_ is the water density, also calculated experimentally with the pycnometer. The composite samples used were obtained after the injection molding process.

## 3. Results and Discussion

### 3.1. Flexural Properties of PA11-SGW Composites

The results of the flexural tests are shown in Table 1, where *V^F^* is the fiber volume fraction regarding the total volume in the composite, *ρ^C^* is the experimental values of the composite density, σ_f_*^C^* is the flexural strength, σ_f_^m*^ is the flexural strength of the matrix at the maximum composite strength, *D* is the experimental deflection of the tested materials, ε_f_*^C^* is the strain of composites at the maximum flexural strength, and *U*_r_ is the resilience of the composites regarding the fiber content in weight percentage (*w*/*w*) in the composite material. ε_f_*^C^* was measured as ε_f_*^C^* = (6*·D·d*)/*L*^2^, were *d* is the specimen depth and *L* is the length of the support span. The contribution of the matrix σ_f_^m*^ was obtained by a curve fit of the experimental stress and deflection values. The values returned by the equation agreed with the experimental data with very low deviations that did not impact the results of the mathematical operations.

The σ_f_*^C^* of the composites increased linearly up to 60% *w*/*w* SGW contents, obtaining a linear fitting with the following equation: σ_f_*^C^* = 123.11*·V^F^* + 38.44, and a correlation coefficient (*r*^2^) of 0.99. The increases of the flexural strength of the composites with the addition of 20 to 60% *w*/*w* SGW, compared to the matrix, were 37.5%, 71.7%, 93.7%, 131.5%, and 156.7%, respectively. It is reported in the literature that such linear behavior is indicative of an optimal interface between the fibers and the matrix as well as a good dispersion of the reinforcements in the matrix [3,45,46]. When the tensile strength of the same composites was investigated, a maximum tensile strength was found for 50% *w*/*w* SGW content, and further increases of the reinforcement content produced a noticeable decrease in this property [38]. One possible explanation for this phenomena could be a poor wetting process of the fibers for high reinforcement contents or the creation of fiber bundles [47,48]. The results show that the effect of such phenomena on the flexural strength is less apparent than in the tensile strength. In short-fiber-reinforced composites, the strength property depends on the fiber and polymer natures and volumes, the fiber morphology and orientation inside the matrix, and the interaction between the fiber and the matrix [49]. These interactions allow for matrix-fiber stress transfer and can be determined by the interfacial shear strength (IFSS). The IFSS, together with the fiber’s orientation, are the most important factors in the strength performance of the composites. Nonetheless, the orientation of the fibers is highly dependent of the mold in injected-molded samples and it cannot be modified. Thus, IFSS could be considered as the main factor of the reinforcement effect of the fibers in the composite strength. In turn, the IFSS is affected by the properties of the reinforcement fiber, the type of bond established between the polymer and the fiber, and the quantity of these bonds [50].

**Figure 2:**
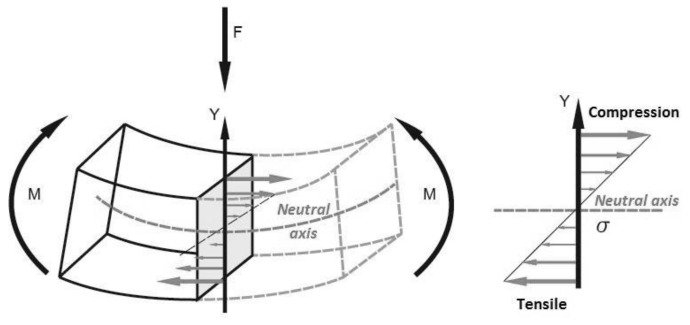
Scheme of load forces acting during the bending test.

A higher enhancement of the flexural strength than that observed for tensile strength of the same composites has been observed [38]. This difference between tensile and flexural strength enhancements is related to the specimens working at tensile and compression loads during the bending test as opposed to pure tensile loads (Figure 2).

During the bending test, the load force is produced in a perpendicular direction over the surface of the sample bar. This force produces a response in the extreme points of the bar to counteract it. The sample response in the vertical axis, where the force is loaded, produced different loads: tensile and compression. Thus, it is expected to find fibers working under tensile or compression stresses. However, as mentioned above, the main factor affecting the stress transmission, and thus the strength property, is the interface. To obtain an enhancement in the flexural strength it is necessary to ensure an optimal interface in the composite material.

**Figure 3:**
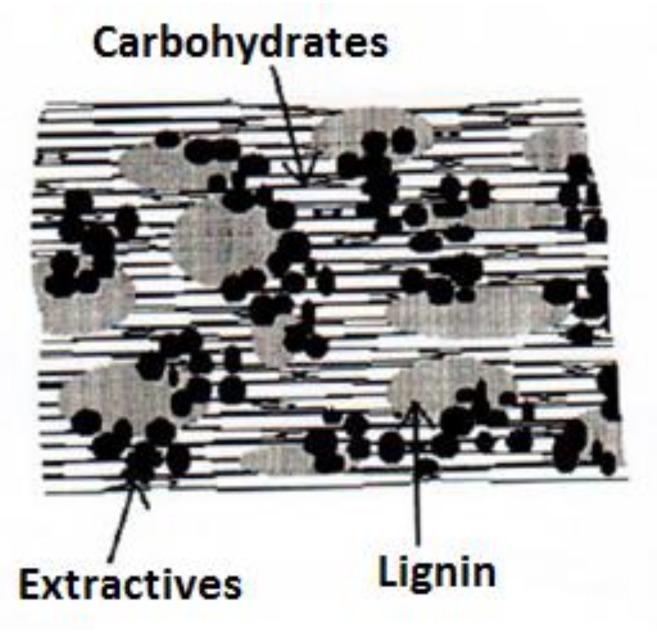
Chemical composition of a lignocellulosic fiber [53].

The flexural strength and its evolution, with respect to the reinforcement content, are related to the formation of a suitable interface. In PA11-SGW composites, this interface is produced by the capacity of the PA11 to interact with the SGW fibers by H-bonds and other intermolecular forces in addition to mechanical anchorage. The SGW fibers used in this work are mechanical fibers from pine, obtained through high-yield processes, and the chemical composition of such fibers (i.e., the carbohydrates, lignin, and extractives) and their distribution along the section is slightly affected by the process. Lignin and extractives are usually found in the most superficial layers of the fiber and can inhibit the interaction between the cellulosic chains and the polymer [51,52]. Böras and Gantenholm [53] proposed a simple schematic model for the chemical distribution on the surface of a mechanical fiber (Figure 3).

**Figure 4:**
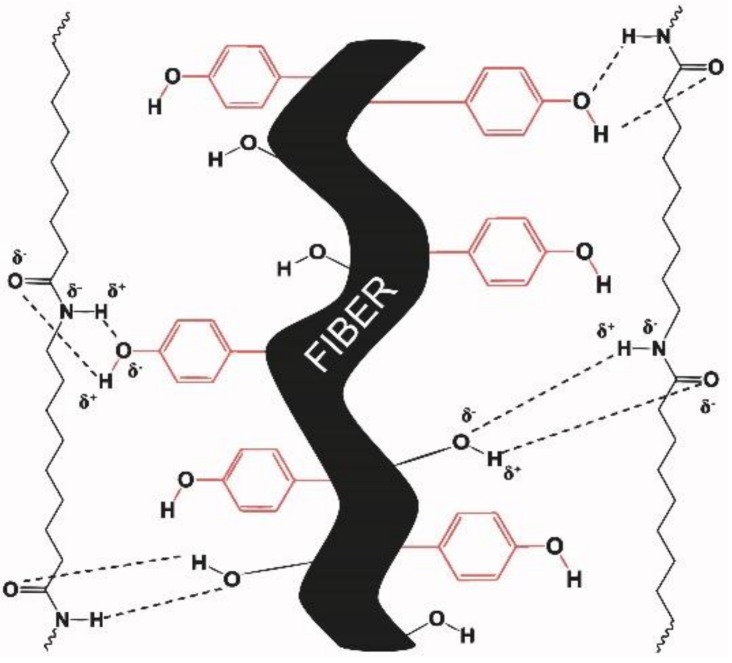
Schematic interaction of PA11 chains with a lignocellulosic fiber. The phenol groups (marked in red) represent the hydroxyl groups provided by lignin and the others represent the hydroxyls groups from the hemicellulose and cellulose.

In this model, the largest area corresponded to lignin (28%) and extractives (32%), and only 40% of the available surface was covered by carbohydrates (cellulose and hemicellulose). In the case of SGW fibers, the cellulose and hemicellulose surface available could be slightly reduced. The lignin content on the surface was expected to be slightly higher than that obtained for thermomechanical treated fibers from softwood [54]. Apart from cellulose and hemicellulose, lignin has also a considerable percentage of hydroxyl groups in its structure, allowing its interaction with the PA11 matrix. A scheme of the interface of PA11 and SGW fibers is proposed in Figure 4. The figure describes a fiber surface with hydroxyl groups from lignin (phenol groups) and carbohydrates available at the fiber surface that can interact with the PA11 matrix.

The capacity of the PA11 to interact at the same time with lignin and cellulose hydroxyls could explain the good mechanical performance obtained for PA11-SGW composites [35]. Without this interaction and due to the moderate number of available hydroxyl groups from cellulose and hemicellulose in the surface as a result of the high presence of lignin in the surface (Figure 3), the composite strengths would probably be reduced. Moreover, the lignin content probably contributes to the enhancement of the fiber dispersion in the PA11 matrix, inhibiting the creation of fiber agglomerates [38,55]. Furthermore, the use of SGW fibers reduced the cost and the production time of the composite materials as it was not necessary to submit the fiber to higher energy- and time-consuming processes. In addition, their use agrees with the postulates of green chemistry and engineering proposed by Anastas et al. [56,57].

**Figure 5:**
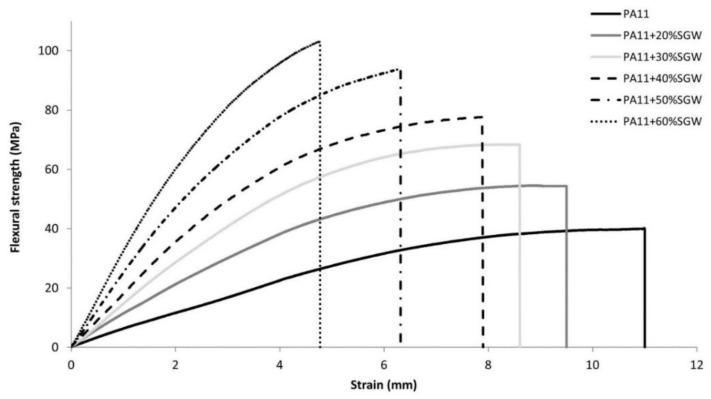
Stress-curves of PA11 and PA11 composites up to the maximum flexural strength of the composites.

Figure 5 shows the stress-strain curves of the composites and the matrix up to the maximum flexural strength. As expected, the deformation of the materials decreased when the fiber content was increased due to the higher stiffness of the fiber. The strain at the maximum flexural strength for the composite with 60% *w*/*w* SGW content was 44% of the matrix. The behavior of the flexural strain of the PA11-based composites was similar to that of the SGW-reinforced PP with the use of a coupling agent [37]. Nonetheless, the values of the PA11-based composites were slightly higher, probably due to the higher strain obtained for this matrix.

A reduction of the σ_f_^m^^*^ and, consequently, of the matrix contribution was observed (Table 1) due to the increasing fiber content, a stiffer phase, and the reduction of the strain of the composite materials [58].

The toughness of a material is understood as the energy which a material can absorb before collapsing. In the case of the flexural toughness of PA11 and PA11 + 20% SGW, the specimens did not collapse during the bending test, so the determination of their toughness was not possible. Nevertheless, the resilience, understood as the ability of a material to absorb energy and release it without suffering permanent deformation, was calculable for all tested materials. The resilience was calculated as the area under the linear or elastic zone of the stress-strain curve. The highest resilience was obtained for PA11 and decreased as the fiber content was increased. The resilience was reduced by 62%, regarding the matrix, at 60% fiber content.

A linear increment of the density was observed for increasing fiber contents, as was expected due to the high density of the fiber (1.40 g/cm^3^) compared to that of PA11 (1.03 g/cm^3^). The density of PA11-SGW composites was slightly higher than that of PP-SGW composites, which was attributed to the higher density of the PA11 matrix compared to that of PP (0.905 g/cm^3^) [59]. In natural fiber-reinforced composites, the polymer density is a key factor due to the similar densities of natural fibers [60,61]. The density of SGW was in the range of other natural fibers, as reported in the literature [15,62], and significantly lower than GF (2.45 g/cm^3^).

### 3.2. Analysis of the Flexural Strength: Fiber Flexural Strength Factor and Average Fiber Intrinsic Flexural Strength

**Figure 6:**
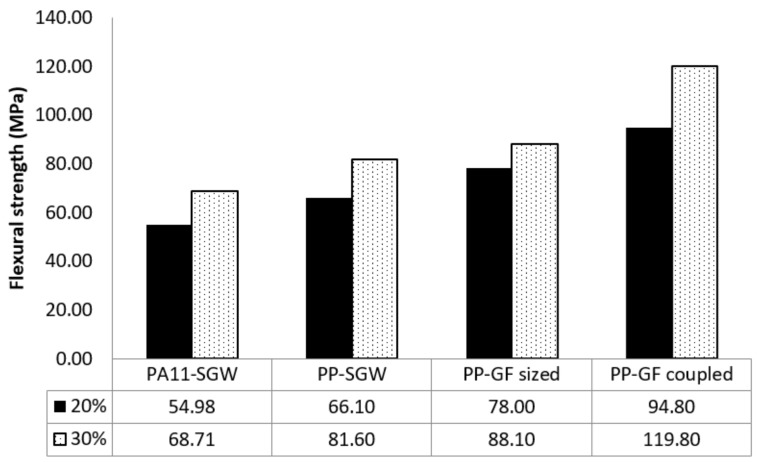
Comparison between PA11- and polypropylene (PP)-based composites at same the fiber content.

In order to assess the competitiveness of PA11-based composites, it is important to compare their flexural strength with those commercially available materials, such as PP-GF and PP-natural fiber composites. PP-GF composites, processes by injection-molding, are usually reinforced up to 20–30% *w*/*w*. In the Figure 6, the flexural strength of PA11-SGW was compared with the values obtained in previous works for PP-SGW [37] and PP-GF [63] composites at the same fiber contents (20–30%). The difference between PP-GF-sized and -coupled composites is the use of GF surface modifications in the case of GF-sized composites and the addition of a coupling agent in the formulation of coupled composites.

A noticeable gap between the flexural strengths of all of the composites reinforced with a 30% *w*/*w* was observed, especially when compared with the GF-coupled reinforced PP composite. The composites reinforced with 30% *w*/*w* of SGW exhibited flexural strengths that were 43% and 32% lower, for PA11 and PP matrices, than the GF-coupled reinforced composite.

**Figure 7:**
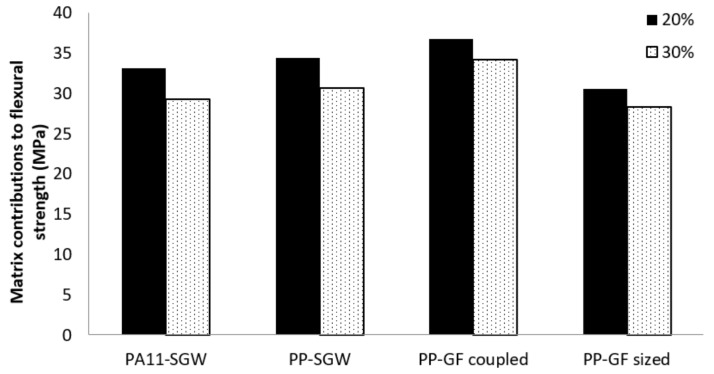
Matrix contribution of PA11-SGW and PP-based composites with 20% and 30% fiber content.

The analysis of the flexural strength behavior can help the understanding of the differences between reinforced PA11- and PP-based composites. The use of a model such as the modified rule of mixtures (mRoM) was proposed to model the behavior of the flexural strength of the composites (Equation (2)) [64].

(2) $$\sigma_{f}^{C}=f_{c}^{f}\cdot V^{F}\cdot \sigma_{f}^{F}+\left(1-V^{F}\right)\cdot \sigma_{f}^{m*}$$

where σ_f_*^F^* is the average fiber’s intrinsic flexural strength and *f*_c_*^f^* is the flexural coupling factor. The flexural coupling factor incorporated the impacts of the mean orientations of the fibers, the morphology of such fibers, and the quality of the interface. In fact, the flexural coupling factor is defined as the product of an orientation factor and a length and interface factor (*f*_c_*^f^* = *X^f^*_1_*·X^f^*_2_). The mRoM models the flexural strength of a composite as the sum of the contributions of the reinforcement and the matrix. Figure 7 shows the contributions of the matrix ((1 − *V^F^*)*·*σ_f_^m*^) to the flexural strength of reinforced PA11- and PP-based composites.

It was found that the matrix contribution values were all in similar ranges. Thus, the f_c_*^f^·*σ_f_*^F^* term of the mRoM (Equation (3)) was responsible for the main differences between the flexural strengths of the composites. In the mRoM, *f*_c_*^f^* and *σ*_f_*^F^* remained as unknown parameters. A fiber flexural strength factor (*FFSF*) was proposed as an useful way to evaluate the neat contribution of the reinforcement to the composite strength [65]. *FFSF* was defined by rearranging the mRoM and isolating these unknown parameters:

(3) $$FFSF=\frac{\sigma_{f}^{C}-\left(1-V^{F}\right)\cdot \sigma_{f}^{m*}}{V^{F}}=f_{c}^{f}\cdot \sigma_{f}^{F}$$

**Figure 8:**
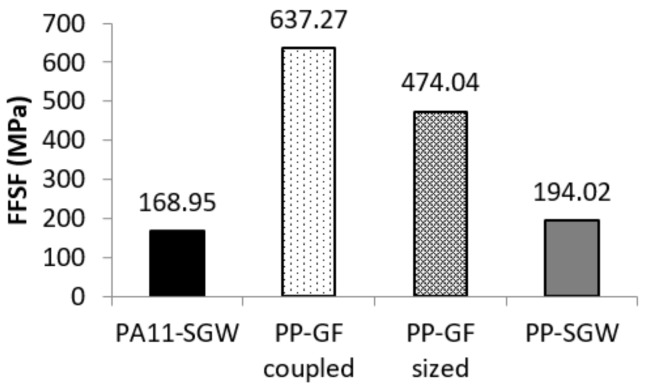
The fiber flexural strength factor (FFSF) of PA11 and PP composites.

In the above, the *FFSF* is the slope of the regression curve obtained from the representation of σ_f_ − (1 − *V^F^*)*·*σ_f_^m*^ vs. *V^F^* [1]. The value of *FFSF* is unique for a family of composites, as its value does not change with the amount of reinforcement due to its dependence on the fiber properties and its interaction with the matrix. The *FFSF* of PA11-SGW composites was calculated and compared with those of PP-based composites obtained from the literature [1,36,63,66] (Figure 8).

The *FFSF* for PA11-SGW was 3.7 and 2.8 times lower than that for PP-GF-coupled and -sized composites, respectively. In the case of SGW composites, a higher value was obtained for the PP-based composites. These differences between SGW-reinforced composites indicated a higher strengthening effect of these fibers in PP. This was also observed between coupled and sized GF-based composites. As mentioned above, *f*_c_*^f^* is dependent on the quality of the interface, the morphological characteristics of the fibers, and their orientation against the loads. Usually, the orientation of the fibers is related to the equipment used to fabricate the specimens via inject-molding, and it was the same for all of the composites. Thus, the differences between the flexural strengths of the composites depend mainly on the fiber properties and the quality of the interface.

The difficulties in measuring the intrinsic properties of the fibers led us to consider the calculation of these properties using theoretical models. One of the methodologies proposed to compute the average intrinsic flexural strength uses the *FFSF* and the *FTSF* (fiber tensile strength factor) [1,37,46]. The proposed relation is σ_f_*^F^* = *FFSF*/*FTSF·*σ_t_*^F^* [1,37]. The ratio between *FFSF*/*FTSF* for PA11-SGW composites was 1.57, which is lower than those obtained for other natural fiber-reinforced thermoplastic composites, with ratios in the range of 1.7–1.9 [1,37,61]. Using this ratio, the computed mean theoretical intrinsic flexural strength of SGW in PA11-based composites was 888 MPa. This value was lower than 1095 MPa, a value found in the literature for SGW as a reinforcement for PP [37]. However, some studies showed that the intrinsic strength of the fibers changes with matrix chemical families [19,46]. In that sense, the value of the intrinsic strength is also a measurement of the exploitation of the strengthening capabilities of the reinforcing fibers. These strengthening capabilities can also be related to the interface type, the quantity of bonds, and their energy value [50]. Because our investigation made use of the same fiber, the differences were related to the interface quality. PP cannot establish strong chemical interactions and a coupling agent was required to do so. The coupling agent used in PP-SGW composites was maleate polypropylene. The maleate part of the coupling agent established covalent bonds and intermolecular forces with the fibers, and its polyolefinic chain became entangled with the polymer matrix [67]. The energetic value of H-bonds may be different depending on the atoms involved and the distance between them, but these bonds are energetically weaker than covalent bonds [68,69]. This explains the lower interface of the PA11-SGW composites in comparison with the PP-SGW composites. However, the presence of this H-bond in the interface produced a difference of 13 MPa, while in the case of uncoupled PP-SGW composites the difference was around 30 MPa [37].

The literature showed that the strengthening effect of wood fibers on polyamide 6 (PA6) composites was also lower than that observed in PP-based composites [30,70,71]. All of these arguments reinforce the hypothesis of a good but not optimal interphase in PA11-based composites. Nevertheless, these intrinsic flexural strengths values were obtained using a back-calculation and the results obtained may differ from experimental results [72].

The intrinsic flexural strengths of GF, sized and coupled, were evaluated at 3787 and 4237 MPa, respectively. These values were 4 times higher than those achieved using SGW as a PA11 reinforcement. Nonetheless, the *FFSF* of the same reinforcements was only 3 times higher. According to the definition of *FFSF* (Equation (4)), these differences were probably related to the values of the coupling factors.

### 3.3. Flexural Strength Performance of PA11-SGW Composites versus Oil-Based Composites

The coupling factor can be considered as an indication of the quality of the interface, the morphological characteristics of the fibers, and their orientation against the loads in the composite. Once the σ_f_*^F^* was obtained, the use the mRoM to calculate *f*_c_*^f^* for PA11-SGW composites was possible. In a previous work, the mean coupling factor for the tensile strength (*f*_c_*^t^*) was evaluated at 0.186 [38]. Using the σ_f_*^F^* obtained in the relation between the *FFSF* and *FTSF*, the coupling factor for the flexural strength was computed to a mean of 0.183. The tensile and flexural coupling factors were very similar, showing that the differences between the tensile and flexural tests had little effect in such parameters [37,66,73]. The same coupling factor computed for the 50% *w*/*w* SGW-reinforced PP coupled composite materials was computed to be 0.173, which was slightly inferior to that of the PA11-based composites (Table 2). This lower result could be related to the lower orientation factor (*X^f^*_1_) found in PP-SGW composites [36,38] as *f*_c_*^f^* = *X^f^*_1_*·X^f^*_2_ and assuming no differences obtained between the flexural and tensile strength’s *X^f^*_1_. This assumption was made because the orientation of the fibers in the composed material mainly depended on the injection-molding equipment employed in the fabrication process [1].

As mentioned above, GF is more prone to decrease its length as the fiber content increases. This impact was reflected when the coupling factor for the sized GF-reinforced PP composites decreased by 18% when the reinforcement contents were changed from 20 to 30% *w*/*w*. In the case of the coupled GF-based composites, this decrease was 9%. Despite its high intrinsic flexural strengths, GF was penalized due to its fragility. Finally, the literature agrees on values of *f*_c_ around 0.2 as an indication of a well-bonded system for semi-aligned short-fiber composites [38,74,75]. The mean *f*_c_*^f^* for PA11-SGW composites was near to this value, indicating a quite good interface.

After the study of the intrinsic properties of the fiber and its interaction with the matrix, a lower reinforcement effect was observed in PA11-SGW compared to PP-SGW composites. This difference was mainly attributed to the lower energetic interface bonds in the PA11-SGW composites. Thus, the interface obtained in both composites was mainly related to the interactions between the polymers and the fibers. In PP-GF composites, the GF surface was modified to obtain a hydrophobic behavior that enhanced the dispersion and produced some interaction between the fiber and the polymer. In GF-coupled composites, the mechanism was the same as that detailed above for SGW fibers. Although the strength of the GF-based composites was higher due to the higher mechanical performance of GF, the sized GF-based composites showed an interface with scarce and probably quite low intermolecular interactions, obtaining flexural strengths slightly higher than PP + 30% SGW and 31 MPa lower than coupled GF-based composites. These results are in agreement with the values of *f*_c_*^f^* observed in both GF composites.

**Figure 9:**
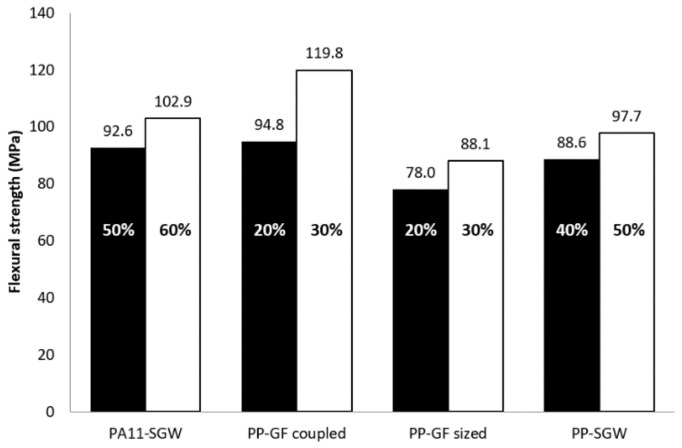
PA11-SGW and PP-SGW composites with high fiber contents versus PP-GF composites.

Finally, Figure 9 compares the flexural strength performance of SGW-based composites with higher reinforcement contents vs oil-based composites.

It was found that the 50–60% SGW-reinforced PA11 composites compared well with the remaining composites, excluding the coupled 30% GF-reinforced PP composite. In fact, to obtain the same flexural strength as the PA11-based composite, a 0.66 volume fraction of SGW was needed. This volume fraction corresponds to a fiber content of around 73%. For PP-SGW composites, the *V^F^* required is 0.583, along with a fiber content of 65% [37]. In both cases, these fiber fractions cannot be achieved due to the bad dispersion and poor wettability of the matrix over the fibers. Moreover, the strength of these hypothetical composites is directly related to the fiber due to the low volume of the matrix and the high stiffness and content of fibers, which drastically reduce their strain at break. Nonetheless, the PA11 + 50% SGW composite can replace almost all of the compared materials (Figure 6). Moreover, the strains of SGW-reinforced composites were similar to or higher than those achieved for PP-GF composites, although the fiber volume fractions were at least 3 times higher [8,37]. These results displayed the suitability of PA11-SGW composites to replace commercial materials as a greener and more sustainable alternative. Furthermore, nowadays PA11 is comparatively quite expensive, but its capacity to be reinforced with high fiber contents in composite materials will reduce drastically the cost of these materials and enable the achievement of competitive commercial prices.

## 4. Conclusions

Fully bio-based composites from bio-based PA11 and high-yield mechanical SGW fibers were successfully formulated and prepared. The PA11-SGW composites were characterized by means of a three-point bending test, as well as calculation of the *FFSF* and the average σ_f_*^F^*. Their competitiveness, in terms of flexural performance, was evaluated against three different oil-based composites: PP-GF coupled and sized, and PP-SGW. The use of a bio-based matrix such as PA11 reinforced with SGW instead of oil-based composites followed the principles of green chemistry.

The flexural strength of PA11-SGW composites evolved linearly up to a maximum value of 102.7 MPa, when 60% SGW content was added to the composite. The PA11-SGW composites with high fiber content (PA11 + 50% SGW and PA11 + 60% SGW) were shown to be able to replace some oil-based composites. Moreover, the strain values of all PA11-SGW composites remained higher than those of the oil-based ones. The competitive results of the PA11-SGW composites and the linear evolution of the results indicated a good interface between PA11 and SGW fibers. This interface was obtained without the modification of any phase and without the use of coupling agents, due to the capacity of PA11 to establish H-bonds.

The strengthening capabilities of SGW fibers were studied using the *FFSF*. SGW showed a lower performance in PA11 than in PP. The differences between SGW-based composites were related to a lower exploitation of the strengthening capabilities of the fiber in PA11-SGW due to a weaker interface (H-bonds versus covalent bonds and entanglement). This weaker interface was also reflected in the lower σ_f_*^F^* of SGW fibers in PA11-SGW compared to that obtained in PP-SGW composites. The slightly higher value of *f*_c_*^f^* of PA11-SGW composites than PP-SGW was related to the higher orientation factor in PA11-SGW. On the other hand, the differences from GF were mainly related to its higher σ_f_*^F^*. Moreover, in the case of the GF-coupled composite, the high FFSF value was obtained by the combination of the GF’s σ_f_*^F^* and the effect of the coupling agent.

Finally, GF reported higher strengthening capabilities than SGW. This implies the necessity of increasing the *V^F^* of SGW up to 3.7 times to achieve a similar fiber contribution to that achieved by GF coupled in the composite strength. However, the use of GF as a reinforcement is limited by its fragility. In terms of processability and sustainability, SGW fibers, especially as reinforcement of PA11, provide better options.

## Figures and Tables

**Table 1:** Flexural properties of Polyamide 11-Stone Groundwood fibers (PA11-SGW) composites.

Fiber content (% *w*/*w*)	*V^F^*	*ρ^C^* (g/cm^3^)	σ_f_*^C^* (MPa)	σ_f_^m*^ (MPa)	*D* (mm)	ε_f_*^C^* (%)	*U*_r_ (KJ/m^3^)
0	0.000	1.03	40.0 ± 1.52	40.0	11.0 ± 0.32	7.39	78.18
20	0.155	1.09	55.0 ± 2.22	39.2	9.5 ± 0.51	6.39	57.77
30	0.240	1.12	68.7 ± 1.79	38.5	8.6 ± 0.45	5.78	49.65
40	0.329	1.15	77.5 ± 1.28	36.8	7.8 ± 0.38	5.24	42.73
50	0.424	1.18	92.6 ± 3.12	32.3	6.3 ± 0.51	4.24	37.05
60	0.524	1.22	102.7 ± 4.75	26.5	4.8 ± 0.47	3.23	29.79

**Table 2:** Coupling factor for the flexural strength.

Composite	Fiber content	*V^F^*	σ_f_*^F^* (MPa)	(1 − *V^F^*)·σ_f_^m*^ (MPa)	*f*_c_*^f^*
PA11-SGW	20%	0.155	888	33.16	0.159
	30%	0.240	888	29.29	0.185
	40%	0.329	888	24.69	0.181
	50%	0.424	888	18.82	0.196
	60%	0.524	888	12.59	0.194
PP-SGW	50%	0.404	1095	20.98	0.173
PP-GF_sized_	20%	0.084	3787	30.60	0.137
	30%	0.136	3787	28.31	0.116
PP-GF_coupled_	20%	0.084	4237	30.60	0.163
	30%	0.136	4237	28.31	0.150
